# Social STEAM Learning at an Early Age with Robotic Platforms: A Case Study in Four Schools in Spain

**DOI:** 10.3390/s20133698

**Published:** 2020-07-01

**Authors:** Elena Jurado, David Fonseca, Jorge Coderch, Xavi Canaleta

**Affiliations:** 1 GRETEL–Research Group on Technology Enhanced Learning, La Salle Campus Barcelona, Universitat Ramon Llull, 08022 Barcelona, Spain; david.fonseca@salle.url.edu (D.F.); xavier.canaleta@salle.url.edu (X.C.); 2 Department of Engineering, Blanquerna, Universitat Ramon Llull, 08022 Barcelona, Spain; jorgeca@blanquerna.url.edu

**Keywords:** educational robotics, elementary education, KIBO robot, STEAM, teacher education, human–robot interaction

## Abstract

Robotics is one of the key learnings in a world where learners will interact with multiple robotic technologies and operating systems throughout their lives. However, school teachers, especially in the elementary and primary education stages, often have difficulties incorporating these tools in the classroom. Four elementary teachers in three schools in Catalonia were trained to introduce robotics in the classroom to seventy-five students. The main actions consisted in classroom accompaniment by a university-trained support teacher, curricular materials’ development, and assessment of the students’ and teachers’ learning. The designed contents and evaluation criteria took into account the potential of educational robotics to improve soft skills and to promote Science, Technology, Engineering, Arts, and Mathematics (STEAM) interdisciplinary learning. Teachers perceived the training to be supportive and useful and ended the school year feeling confident with the used robotic platform (KIBO). The assessment of the students’ learning showed an average mark of 7.1–7.7 over 10 in the final evaluation criteria. Moreover, students’ learning was higher in the classes where the teachers had higher initial interest in the training. We present and analyse the actions carried out, with a critical and constructive look at extending the experience to other educational centers.

## 1. Introduction

Learning robotics forms part of the core learning skills in a world where students have to engage with multiple robotic technologies and operating systems throughout their lives [1,2]. In a robotics class, students construct and program robots, practice engineering and computational thinking [3], and develop logical-mathematical and problem-solving abilities. The tangibility of robotic platforms is a decisive factor in the motivation of students, as it allows a better immersion in the learning process [4,5]. Robotic platforms have also proven to be a very effective means of learning Science, Technology, Engineering, Art, and Mathematics (STEAM) interdisciplinary knowledge [1] and of developing do-it-yourself projects in schools in the frame of the Maker Movement [6]. Additionally, soft skills like creativity, collaboration, communication, autonomy, and overcoming failure can benefit from didactic units with robotic platforms [7,8]. Further STEAM-related benefits such as cooperative learning and the development of projects that design solutions to social problems can be acquired through educational robotic activities [9]. Therefore, it is crucial that educational robotics forms part of the landscape of educational tools and methodologies in schools and become a key element in the union and articulation of interdisciplinary learning.

Previous studies have shown that students have rich learning experiences in robotics with a learn-by-doing approach, constructing their own robots and programming them with scaffolds [10,11], with open-ended challenges [12], and without top-down instruction but with a structured guided discovery [13]. Strategies such as having students working on part-complete solutions, game-strategy creation, and pair programming have been reported as beneficial, specially for weaker students [10]. Moreover, intervention approaches, such as asking students to reflect on their experience programming and constructing the robot and allowing time after constructing benefited students’ practices [14].

Early childhood is a critical developmental period for learning and therefore an excellent opportunity to incorporate robotics and other new technologies into curricular activities [15]. It can offer unique ways of fostering peer-to-peer interactions and computational thinking [16]. In early childhood settings, robotic manipulation allows children to participate in creative explorations, develops fine motor skills and hand-eye coordination, and engages in collaboration and teamwork [17,18]. The visualization of the output of the programming code, either via the performance of a robot or via a multisensory approach, and unplugged computational thinking activities help students to understand difficult computing concepts [8,19]. However, students’ learning of robotics in naturalistic classroom settings, at early ages, and related with other contents is still not well-understood, and there is a need to conduct studies in this area [20].

Teachers need a deep understanding of computer science concepts and pedagogical practices to be able to design robotic experiences and assessments that truly enable students to acquire computational thinking and the development of soft-skills [2]. Traditional methods of technology training for teachers such as courses and workshops are ill suited to producing the “deep understanding” required for effective learning outcomes in robotics [21]. As a result, in many cases, robotic educational activities simply reinforce instructionist ways of teaching and learning, focusing only on the tool and not on the concepts, and are unrelated to the the rest of the contents [7]. At the moment, standard techniques of teacher professional development for elementary, primary, and secondary schools are self-contained and divide the acquisition and the application of skills. They generally consist of a series of sequential standalone courses and workshops, concentrated in the first fortnight of July (end of the academic year), where teacher availability is low (imminent vacations) and fatigue at the end of the course is high. It is essential to offer teacher opportunities for professional development in robotics with a learn-by-doing approach, spaced out during the academic year, and with a program that incorporates coaching or teacher partnerships [16,22,23].

Elementary teachers, with the challenge of being knowledgeable in many subjects and with a general lack of an adequate technological training during the course of their teacher education program, do not usually have genuine knowledge of technological and robotic tools [18]. An inadequate access to supporting materials and instructional support, a lack of familiarity with the constructionist methods employed to engage students in robotics engineering design challenges, a lack of preparation time, and a lack of knowledge in making connections between robotics and other subjects have been reported [12]. Although children are growing up in an increasingly digital and technological environment, school curricula do not always focus on exploring the technological world, especially at early ages. In the described context, teacher accompaniment in robotics at elementary schools linked with quality external advice can be key to incorporating robotics into the school curriculum.

La Salle Ramon Llull University in partnership with Pedagogia La Salle Catalunya has recently started EduEnginy, a project that promotes computational and engineering thinking, problem-solving, and STEAM through robotic technology. In the framework of the EduEnginy project, a roadmap has been created to ensure that robotics is adopted in compulsory education curricula in all the schools of La Salle in Catalonia before 2021. The main actions consist in classroom accompaniment and development of curricular materials. It benefits from the experience gained in the pilot test during the 2016/2017 school year [24]. The objectives of this study are thus (i) to present a case study to train elementary teachers to introduce robotics in the classroom by forming partnerships of elementary teachers with external university-trained support teachers that highlight the social and STEAM potential of educational robotics, (ii) to analyse the learning of teachers and students during this process, and (iii) to present future lines for implementing the developed methodology of teachers’ training to other schools.

## 2. Materials and Methods

### 2.1. The Used Robotic Kits

**Figure 1:**
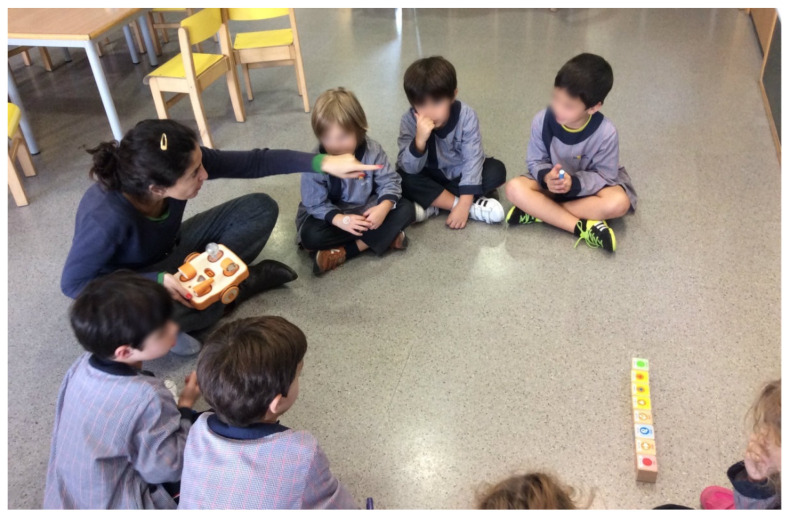
Teaching students with the KIBO robot.

The interventions at the educational centers were implemented using the KIBO robot 18 Kit (https://kinderlabrobotics.com/kibo/). Each school acquired 1 to 4 units of this educational robot, which were then used in groups of 4 to 15 students. KIBO (Figure 1) is the result of 15 years of research at KinderLab Robotics at Tufts University and the evolution of its ancestor KIWI [25]. It is a robotic kit designed for children 4 to 7 years that allows tangible programming without a computer and without the need for reading comprehension. Children make programs through the concatenation of wooden cubes, each one representing an instruction and with pictograms marked with barcodes on the faces. Subsequently, children scan the sequence of instructions with the barcode reader in the body of the robot and KIBO acts according to the given instructions. KIBO also allows the incorporation of various types of sensors (light, sound, and distance) and actuators (motors and light bulb). Its tangible programming language supports parameters, loops, and conditions.

The scientific foundation behind this tool as well as its robustness and the wide range of pedagogical activities around the learning of interdisciplinary STEAM that it allows were decisive for using it in this study [25,26]. Its programmable wooden blocks emulate traditional Montessori tangibles and support a collaborative student environment [17]. On the other hand, the combination of tangible programming and robot construction available with KIBO offers unique opportunities for educational robotics that are not present in other popular educational robots at early ages such as Bee-Bot [15,27]. Likewise, we see the benefit of having manipulative programmable building bricks, which would make KIBO a better option than tablets or computer screens at early ages [5,19,28].

### 2.2. Methodology to Train Teachers

The early childhood teacher training was implemented to 4 teachers in 3 elementary La Salle schools in Catalonia (Spain). They were accompanied throughout the course in 2017/2018 while teaching to 5 different groups of 15 students (65 children 4–6 years old). After an initial meeting and presentation with all the elementary stage coordinators of all schools of La Salle Catalonia, the management teams of various schools asked to receive the training, motivated but not obliged by the pedagogy team of these schools’ network.

**Figure 2:**
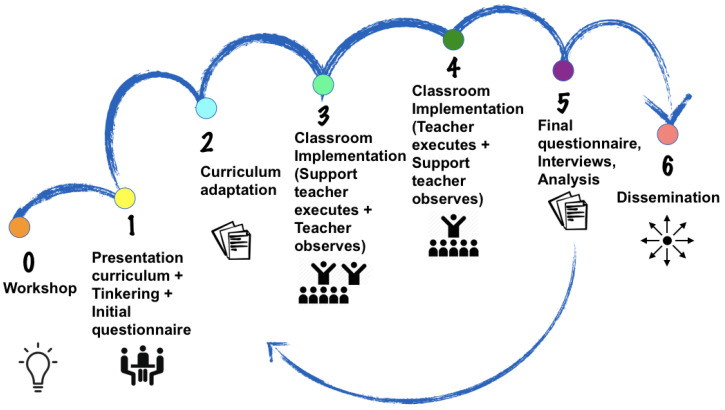
Methodology of the teachers’ training.

The teacher training was structured according to the phases proposed in Figure 2.

After a curriculum adaptation to the school idiosyncrasy, sixteen 45–60 min long sessions were designed for a period of 8 months starting in fall. The training started with a three-day teacher professional development workshop in the summer before the implementation, which covered the essentials of robotics in early education. It followed a two-hour workshop in each school focused on the presentation of the curriculum and on an initial questionnaire to the teachers (Appendix A and Appendix B). During the school year and every two sessions, the university-trained support teacher accompanied an elementary school teacher in the classroom. The session was taught first by the support teacher (while the trained teacher observed) to one half of the class and then by the trained teacher (while the support teacher observed) to the other half of the class. The support teacher was in fact a trained faculty member with expertise in research on pedagogy and robotics. In the course of the sessions, written documentation with the sequence of teaching-learning activities was delivered to the teaching staff and a post-class online support was delivered. At the end of the academic year, all teachers were asked to complete a final questionnaire (Appendix C) and to design an extra session. Then, the most talented teachers were selected to be instructors for other teachers in the following year summer workshop.

In summary, the training of this study was designed to allow early childhood teachers to learn-by-doing, that is, to learn about robotics while teaching their students. It favoured the acquisition of technological skills when the teacher truly needed them (while teaching), avoiding standalone courses that divide between how and when skills are learned. This approach is supported by the TPCK (Technological, Pedagogical, Content Knowledge) model as a framework for good teachers in the 21st century [21]. Teachers in this study were also trained to support the TPCK framework by promoting activities for teaching technology that honor the rich connections between technology, the subject matter (content), and the means of teaching it (the pedagogy).

### 2.3. Developed Curriculum

While training the elementary school teachers, a curriculum was designed adapted to the needs of the educational stage. The designed curriculum consisted in 16 sessions lasting between 45 and 60 min, organised around computer science concepts introduced along the way, such as instruction, sequence, iteration, and conditional. Moreover, each of the sixteen sessions had activities related to interdisciplinary STEAM learning and were closely inspired by the results in the previous pilot experience [24] and by scientific literature related to the robotic tool [16,26]. Thanks to the good results in the teachers’ perceptions and in the students’ learning in the pilot experience, we advocated for a similar constructionist-based problem-solving curriculum, anchored in problems relevant to the young learners and with scaffolding and reflection activities.

The use of KIBO robots in the curriculum not only included the technology but also integrated it into the teaching target content. As such, robotics was used for interdisciplinary learning in all the STEAM subjects: science (sessions 3, 9, and 11), technology (sessions 1 through 15), engineering (sessions 1, 2, 5, 6, 14, and 15), arts (sessions 2, 5, 6, 7, 14, and 15), and mathematics (sessions 7 and 8). Moreover, the curriculum was developed using the Positive Technological Development (PTD) framework [29] recommended for educational programs that use new educational technologies, such as KIBO robotics. The PTD framework adds psychosocial and ethical educational components to the traditional cognitive ones in computer literacy and technology subjects. It encourages six behaviors in students: communication, collaboration, community building, content creation, creativity, and choice of conduct. Those behaviors were encouraged in the developed curriculum.

The sessions were taught to classes of 15 students, following a similar structure in all of them: (1) introduction or reinforcement of concepts, (2) challenge with the robot, (3) technology circle to reflect on the learning received, and (4) free exploration. The challenges with the robot were performed by dividing the students into heterogeneous groups of 4, with interchangeable roles of “builders”, “programmers”, and “responsible for the pieces”. The titles and short descriptions of the sessions of the developed curriculum are presented in the Table 1. The detail of the sequence of teaching-learning activities of an example session together with the related learning objectives is shown in Appendix A.

### 2.4. Methodology to Evaluate the Training

The training for the schools has been evaluated with a mixed-method research approach. This method is based on a pragmatic paradigm that contemplates the combination of quantitative and qualitative items to evaluate teachers’ and students’ learning. It is is suitable for studies for small samples (less than 10), such as ours, and has successfully been applied in previous studies in educational technology-rich contexts [30]. The quantitative aspect of the approach consists of questionnaires for the teachers and of students’ learning assessments. The qualitative aspect of the approach consists of observations of the performance of the instructors during the sessions, of an interview with all the teachers at the end of the academic year, and of remarks from the teachers in the post-class online support.

In summary, we have used the following items:

- Items to evaluate the teachers’ learning: Questionnaires for the teachers at the beginning and at the end of the training (initial and final questionnaires available in Appendix B and Appendix C), observations of the performance of the teachers during the sessions (based on observations of their enthusiasm and of their computational and pedagogical skills during the classroom support), an assessment of the session designed by each teacher at the end of the training, an interview with all the teachers at the end of the training, and evaluation of the remarks from the teachers in the post-class online support via instant messaging.
- Items to evaluate the students’ learning: Analysis of students’ learning assessments by means of a five-point likert-type scale with a list of evaluation criteria based on the PTD (Positive Technological development Engagement Checklist) [29], derived from the integration of ethical and psychosocial components into the cognitive components traditionally used in the evaluation of learning in areas of computer science and technology. We adapted the list of evaluation criteria into five categories: communication (CA1), collaboration (CA2), content creation (CA3), creativity (CA4), and choice of conduct (CA5) (each category is described in Appendix D), and each evaluation criterion had the same weight in the final grade. The students’ learning assessments took place at various control points throughout the course: an initial assessment based on the performance during the programmer’s game at session 3 and on the initial drawing of the robot at session 2, a mid-term individual test at session 10, and a final assessment of the robotic challenge using workstations at session 15.

The evaluation criteria used in this study relate closely to the evaluation criteria, the so-called capacities, established by the local Catalan curriculum of elementary schools [31]: learn to act autonomously (related closely to CA1 and CA5), learn to think and to communicate (related closely to CA1 and CA3), learn to discover (related closely to CA4), and learn to live the world (related closely to CA1 and CA2).

## 3. Results and Discussion

### 3.1. Teachers’ Learning

Taking as reference the interaction of the school teachers with the support teacher during the training, school teachers proved to be communicative, especially in the face-to-face sessions. Stage coordinators adopted a very supportive attitude as well, building bridges with the management team and actively intervening in the dissemination of the experience in different media (http://www.torreforta.lasalle.cat/la-robotica-ja-forma-part-de-les-activitats-curriculars-dels-nens-i-nenes-de-p5-de-la-salle-torreforta/, https://figueres.lasalle.cat/programes/). Moreover, the attitude of the management team from each school was decisive for the training. The initial interest of the teachers responded closely to the positive attitude of the director of the school regarding the promotion of robotics and STEAM. This finding agrees with Thibaut et al. [32], who showed that management support is the most important factor of school context to integrate STEAM education in the curriculum.

**Figure 3:**
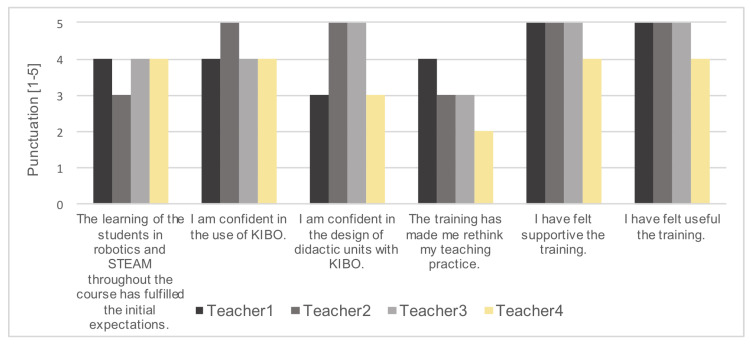
Results of the final questionnaire, where each of the statements presented was scored from 1 to 5.

Based on the questionnaires filled out at the end of the 2017/2018 school year (Appendix C), teachers felt that the training was supportive (average of 4.75 over 5, N = 4) and useful (4.75/5) and felt confident with the use of KIBO (4.25/5) or with the design of related sessions (4/5) (Figure 3). All teachers scored similar results, regardless of their initial programming skills or their initial interest. Those results are similar to those obtained in the pilot test [24], where results ranged from 3.8 to 5 with a sample of 5 teachers and a similar final questionnaire. In addition, during the training, we observed that the emotional engagement of the teachers with the robot increased, as we saw significant changes in attitude from initial fear and frustration towards confidence and empowerment. We also noticed an interest in teaching at the subsequent summer workshop for the more advantaged teachers.

According to the teachers, learning of the students in robotics and STEAM throughout the school year partly fulfilled the initial expectations (3.75/5). A better explanation of the expectations of the students’ expected learning at the initial meeting with the school could improve this score. Finally, we noticed that the training partly made teachers rethink their teaching practice (3.00/5). Two factors may affect this latter value: (i) it is the first time that the teachers of our study received a training like this and we are aware that it takes time to reflect on a change in their modus operandi, and (ii) the current educational model does not generally favour temporary spaces of reflection to cause significant changes in the teaching practices.

Three teachers out of four designed a final extra session with the robot. The session they designed not only included the technology but also integrated it into the target teaching content. Their designed sessions, though, were efficient but not disruptive; the training was rather a tool to empower them, to become familiar with a methodology to teach robotics according to well-researched methodologies, and to increase their confidence in integrating robotics into their day-to-day practice.

In summary, the school teachers rated having support from EduEnginy and La Salle Ramon Llull University as a valuable factor and started to integrate robotic technologies in their educational practice. These positive results agree with studies which show advantages of using teacher partnerships in the classroom [12,16,23]. The presented methodology is very different from many workshops for teachers condensed at the end of the academic year and where the teacher is rather a passive receiver. In this study, the teachers had to develop their skills in the area of robotics when they most needed it: in the classroom and during the academic year.

### 3.2. Students’ Learning

Evaluation of students’ learning is based on the data collected in five groups of fifteen 4–6-year-old students from three different schools in Catalonia and four teachers (N = 75). The data originated from three control points and used the PTD evaluation criteria as described in Section 2.4 and Appendix D. The data is limited partly because, at early ages, the assessment of learners relies largely on the teachers’ observations and, partly, because it was difficult for the university-trained support teacher to simultaneously measure the learning of the students and of the teachers. The results (Table 2) show an average mark of 6.8 in the test at session 10, an average mark ranging from 7.1 to 7.7 in the final PTD evaluation criteria, and an improvement of all the evaluation criteria with respect to the initial PTD evaluation criteria (averaged increase of the PTD evaluation criteria of 0.1 to 0.3 points over 10). The biggest increase was in conduct (increase of 0.3), followed by creativity, content creation, communication, and collaboration (increases of 0.1). The analysis of the increases of the first and third quartiles of each students’ group (Q1 and Q3 in Table 2) disclose interesting trends: children with lower marks (Q1) improved especially in conduct and creativity and did not improve in collaboration and communication; children with higher marks (Q3), instead, improved in collaboration, communication, and creativity and decreased in content creation. The lower increases in communication and collaboration may be explained as of the evolutionary momentum of children at these ages. They are developing their autonomy, and the work is in general more individual than collective [31]. We should bear in mind, though, that the bias could also be due to an inaccurate initial evaluation of some students who seem very bright at mathematics and to the teacher directly attributing it to talent in robotics. A detailed explanation of such findings is beyond the scope of this paper and would need more evaluation points, more evaluation tools, and more groups assessed, but it indicates a need for greater analysis of the collaboration and interaction among students.

On the other hand, the results show differences of up to two points out of ten in the average of the final assessments of teachers (Table 2) and differences of up to 1.8 points out of ten in the increase of the final with respect to the initial PTD assessment. To understand such differences among teachers, we analysed the correlations between students’ marks (students’ marks on the test at session 10 (S1), students’ marks on the PTD evaluation criteria (S2), and students’ increase on the PTD evaluation criteria (S3)) and various variables related to the school teachers (the observation of the teacher’s enthusiasm and computational and pedagogical knowledge during the training (Tp), the teacher’s initial programming ability (assessed by means of the level obtained in a programming related game—blockly maze game—in the initial questionnaire) (Tb), and the teacher’s initial interest to receive support as expressed in the initial questionnaire (Ti)).

**Figure 4:**
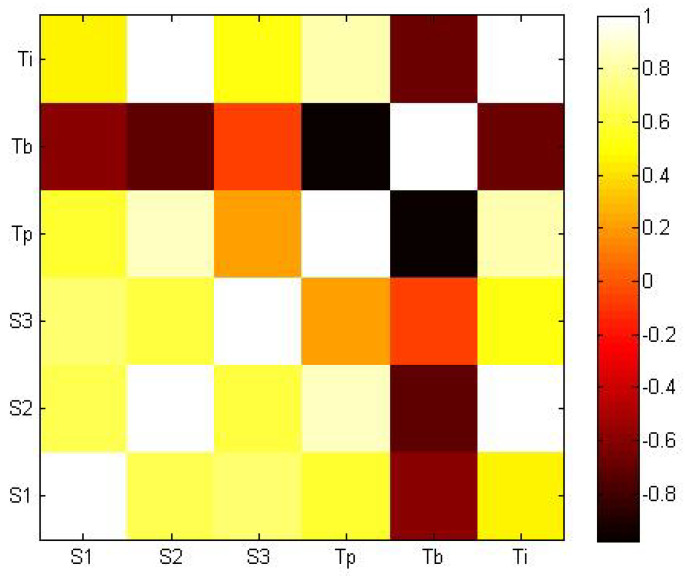
Correlation matrix containing Pearson’s correlation coefficients for each pair of variables: Light colours represent strong positive linear correlations, whereas dark colours document negative correlations. Orange suggests no correlation. Variables correspond to student’s marks in session 10 (S1), student’s final PTD evaluation criteria (S2), student’s increase between final and initial PTD evaluation criteria (S3), teacher’s performance during support (Tp), teacher’s initial level of programming ability (Tb), and teacher’s initial interest to receive support (Ti).

The pseudocolor plot of the correlation coefficients (Figure 4) indicates positive correlations between S1, S2, S3, Tp, and Ti, with the strongest correlation between S2 and Ti (p<0.05). Moreover, Tb and Tp, and S1 and S2 show negative correlations, with Tp and Tb having their negative correlation statistically significant (p<0.05). No dependency between some variables is observed, for instance between S3 and Tp and Tb. We have thus found out that students’ marks were higher for the classes where the teachers showed a greater initial interest in the support. In addition, teachers with lower programming abilities tended to perform better in the support. Those conclusions should be taken with a certain caution due to the limited amount of collected data, but they indicate interesting trends that should be taken into account for future training programmes.

Finally, various important observations were collected from interviews with teachers: (i) robotics helped all the students in their problem-solving abilities; (ii) small groups of 15 students were more suitable than larger groups of 30 students during the robotic session; (iii) psycho-motive and assembly-related activities were successful among the students; (iv) activities involving repeating patterns presented difficulty; (v) collaborative learning was complex to implement since some students monopolised the robot; and (vi) it was complicated for the teachers to assess the initial level of “content creation” evaluation criterion at the beginning of the training. The latter could partly explain the differences of learning increase between Q1 and Q3 students.

The increase of problem-solving skills observed in the students is aligned with findings from prior research indicating that the use of robotics can lead to improvements in problem-solving skills [16,18].

### 3.3. Future Directions

The methodology of teacher training to introduce educational robotics at an early age presented in this study is solid enough to reproduce it in other schools. It has confirmed the good results obtained in the pilot test [24] and has been proven to empower teachers, especially those that have more interest and less initial knowledge, and to benefit student learning. In fact, La Salle Catalonia has agreed to repeat the experience in another 17 schools from its network that incorporate childhood education, and we are analysing the possibility to apply the methodology presented in the article to primary stages in the same network of schools. Additionally, La Salle ARLEP will take as a reference the presented experience to create a roadmap to introduce robotics in early childhood in 110 schools in Spain and Portugal. The aim is to promote robotics in with the same vision: robotics as a tool for interdisciplinary STEAM learning and with an emphasis on promoting ethical and psychosocial components besides computational and engineering thinking.

Keeping in mind the learned experience and the analysis of the results, we suggest two main areas of improvement: improvement in the methodology of training for teachers and improvement in the pedagogical practices with students. Regarding the methodology of teacher training, we suggest allocating more resources to teachers with lower programming abilities and higher initial interest. Even though all teachers felt the training was useful and personalised, students tended to learn more with teachers who had higher initial interest in the training. In the face of future interventions, this initial interest should be encouraged or awakened (or by ensuring that those who participate in the project do so on their own initiative without pressure from the management team of the school). We also suggest the incorporation of other tools to assess the teachers’ subjective experience of the training such as BLA (Bipolar Laddering) or IMI (Intrinsic Motivation Inventories). The practice of selecting the best teachers to teach other teachers at the end of the school year was very beneficial; it empowered the more talented and outstanding teachers. We recommend, thus, to extend this practice. The best teachers would be those who have better integrated didactic and methodological aspects but are not always the best programmers.

Regarding the pedagogical practices with students, we suggest improving the accuracy of the initial evaluation of the students so that it becomes more objective and to introduce more evaluation points and other evaluation tools such as interviews and video recordings. We are keen to rethink the collaborative learning among students. We propose the creation of “corners” or “learning environments” in the classroom, where the children could work in pairs or individually instead of larger groups. The choice of space by the child is free as well as the manipulation of the objects that make up the space. It would be, therefore, a free open space. The teacher could ask the children questions in order to trigger new discoveries.

## 4. Conclusions

We have presented the methodology and results of the training for 4 elementary teachers to 3 schools during the 2017/2018 school year to introduce robotics in the classroom. The main actions consisted in classroom accompaniment by a university-trained support teacher, curricular materials’ development, and assessment of the students’ and teachers’ learning.

Based on the responses from the questionnaires, the acceptance and usefulness of the training were positive. The teachers felt they had learned about the robot (KIBO) and felt competent to create related didactic units. Their performance within the training was more fruitful for those with more initial interested and with lower initial programming abilities, and it also reflected better evaluation results for the students. The presented training methodology is innovative, especially in a context where teachers receive numerous workshops where they tend to act as passive and nonactive learners. The training gives them the possibility to learn by doing. It also supports the TPCK framework [21] by promoting activities for teaching technology that foster connections between technology, the subject matter (content), and the means of teaching it (the pedagogy). On the other hand, the robotic tool used (KIBO) has an important tangible component and enables the design of STEAM interdisciplinary didactic units and the development of soft skills in young learners.

The developed curriculum covered ethical and psychosocial components of the cognitive components traditionally used in the evaluation of learning in areas of computer science and technology. The 16 sessions were designed under the PTD framework [16,29], promoting a holistic training of the students, and encouraged interdisciplinary learning of STEAM. We emphasise the success of psycho-motive and assembly activities among students. Instead, activities involving an abstract use of computational concepts such as repetition presented a certain difficulty.

The results of the final assessments of students showed an average mark of 7.1–7.7 over 10 in the final PTD evaluation criteria and an average increase in all the PTD evaluation criteria of 0.1 to 0.3 points. This increase was not uniform for all students: brighter students tended to improve more in collaboration, communication, and creativity, and those with lower marks tended to improve more in conduct and creativity. Differences among teachers were present, underlining the dependence of their initial interest on their students’ learning. These statements should be analysed with caution because of the limited number of students/teachers assessed (75 students and 4 teachers) and of the uneven evolutionary moments of children at these ages (4–6 years old), but they indicate insightful trends.

The experience presented here points out interesting guidelines to integrate STEAM learning at an early age with robotic platforms to other schools. Regarding the methodology of teacher training, we suggest allocating more resources in teachers with higher initial interest and to extend the practice of selecting the best teachers to teach other teachers at the end of the school year. The best teachers would be those who have better integrated didactic and methodological aspects but are not always the best programmers. Regarding the pedagogical practices with students, we suggest improving the accuracy of the initial evaluation of the students so that it becomes more objective and to introduce more evaluation points and other evaluation tools such as interviews and video recordings. We also encourage new strategies to promote a more similar learning among students. We propose the creation of “corners” or “learning environments” in the classroom, where the children could work in pairs or individually instead of larger groups.

Summarising, it is fascinating to observe how robotics can influence pupils’ learning at early ages, both as a motivating element and as an articulator to promote social STEAM learning.

## Figures and Tables

**Table 1:** Sessions of the developed curriculum: It also indicates the Science, Technology, Engineering, Arts, and Mathematics (STEAM) contents treated during the sessions.

Session	Description	STEAM Contents
S1	Presentation of KIBO. What is a robot? Presentation of the KIBO robot. Students will become familiar with the robot and explore the different parts. In addition, students will play a kinesthetic game to understand that robots are made up of three basic parts (sensors, processor, and actuators).	Technology, Engineering
S2	A drawing of KIBO. What are the rules to take care of the robot? In this session students will make a drawing of KIBO, differentiating sensors, processor, and actuator with different markers. This session will also introduce the rules for taking care of KIBO.	Technology, Engineering, Arts
S3	The robot moves. What is an instruction? What is a program? Through psychomotor skills, students will work on movement sequences and on computational concepts such as sequence, beginning, end, and instruction. Children will program KIBO to represent the movements of an animal. The concept of program will be introduced as a set of instructions.	Science, Technology
S4	I have a robot at home. Review what an instruction and program is. Students will work with the computational concepts introduced in the previous session: sequence, start, end, and instruction. In addition, the KIBO will be contextualized at home, doing something more typical of people: sleeping, bathing, etc. An attempt will be made to translate the drawn action into a series of instructions.	Technology
S5	The parking of KIBO I. What is an engineer? How do we plan a parking lot for KIBO? The goal of this session is to learn what an engineer is and to do the first steps in building a parking lot for the KIBO. A process similar to engineering design will be followed, with the sequence of imagining, creating, testing, and sharing.	Technology, Engineering, Arts
S6	The parking of KIBO II. How do we create a parking lot for KIBO? What programming do we give to KIBO to park it? In this session, the car park for the KIBO will be completed and students will program the KIBO to park it under the parking lots they constructed.	Technology, Engineering, Arts
S7	We make patterns. What is an iteration? Students will construct geometric figures and will make patterns.	Arts, Maths.
S8	KIBO practices patterns and iteration with geometric shapes. In this session, the geometric figures created in the previous session will be used. The students will generate patterns on the floor with geometric figures and KIBO will follow them.	Technology, Maths.
S9	Programming KIBO while doing a tour of the world. Using KIBO and a mural designed for this purpose, the location of the continents will be reviewed. Likewise, the robot will be programmed to go from one continent to another, and kids will practice loops and spatial orientation.	Science, Technology
S10	Student evaluations. There will be a test to evaluate individually the learning of students in the programming of KIBO.	Technology.
S11	What are the sensors? The KIBO responds to claps. In this session, students will learn what the sensors are and how they feel to their senses. The ear of the KIBO, which has a sound sensor, and the associated instruction “Wait for Clap" will be used. The instruction “Wait for Clap" will be used to make the robot turn around by itself.	Science, Technology
S12	Travelling words after clapping. Students will program the robot to chain letters in a grid, forming the desired words and using the ear sensor.	Technology (+ Language)
S13	Let us lighten the KIBO. Telescope sensor, conditional. This session explains the use of conditionals by programming the telescope sensor, which distinguishes far and near. In addition, the instruction of the white, blue, and red light bulbs is introduced.	Technology
S14	Decorate the hat of the KIBO. In this session, students will design a hat for the KIBO with recycled material. The KIBO will be programmed to go around following a background music.	Technology, Engineering, Arts
S15	Individual assessments using workstations. By using corners in the classroom, students will perform various tasks: draw the KIBO, program and assemble the KIBO to achieve a small challenge, free programming, and free assembly.	Technology, Engineering, Arts.
S16	Session designed by the teacher.	Science, Technology, Engineering, Arts, Maths

**Table 2:** Averaged grades for each group of fifteen students during the training (N = 75): The grading scale ranges from 1 to 10, with 10 being the highest grade. The  Positive Technological Development (PTD) evaluation criteria correspond to communication (CA1), collaboration (CA2), content creation (CA3), creativity (CA4), and choice of conduct (CA5). The averaged first and third quartiles of the students’ group (Q1 and Q3) are also presented.

Students’ Group	School	Teacher	Test S10	Final PTD Evaluation Criteria					Increase (Final PTD-Initial PTD)				
				CA1	CA2	CA3	CA4	CA5	CA1	CA2	CA3	CA4	CA5
1	1	Teacher1	8.3	7.1	7.0	7.3	7.4	8.3	0.7	0.6	0.5	0.7	1.5
2	1	Teacher2	3.9	5.9	5.8	6.0	5.8	7.2	−0.2	−0.5	0.1	−0.2	0.1
3	2	Teacher3	7.4	6.3	6.6	6.6	6.5	6.7	−0.4	0.4	−0.2	−0.3	−0.3
4	3	Teacher4	6.3	8.1	7.7	8.0	8.4	8.1	0.1	−0.4	0.0	0.4	−0.1
5	3	Teacher4	8.0	8.5	8.3	8.3	7.8	8.1	0.0	0.2	0.0	0.0	0.3
		**AVERAGE**	**6.8**	**7.2**	**7.1**	**7.2**	**7.2**	**7.7**	**0.1**	**0.1**	**0.1**	**0.1**	**0.3**
		**AVERAGE Q1**	**5.6**	**5.9**	**5.8**	**6.4**	**6.9**	**7.5**	**−0.7**	**−0.4**	**0.2**	**0.7**	**0.9**
		**AVERAGE Q3**	**9.0**	**8.0**	**8.1**	**8.1**	**8.3**	**8.4**	**0.2**	**0.3**	**−0.3**	**0.2**	**0.0**

**Figure A1:**
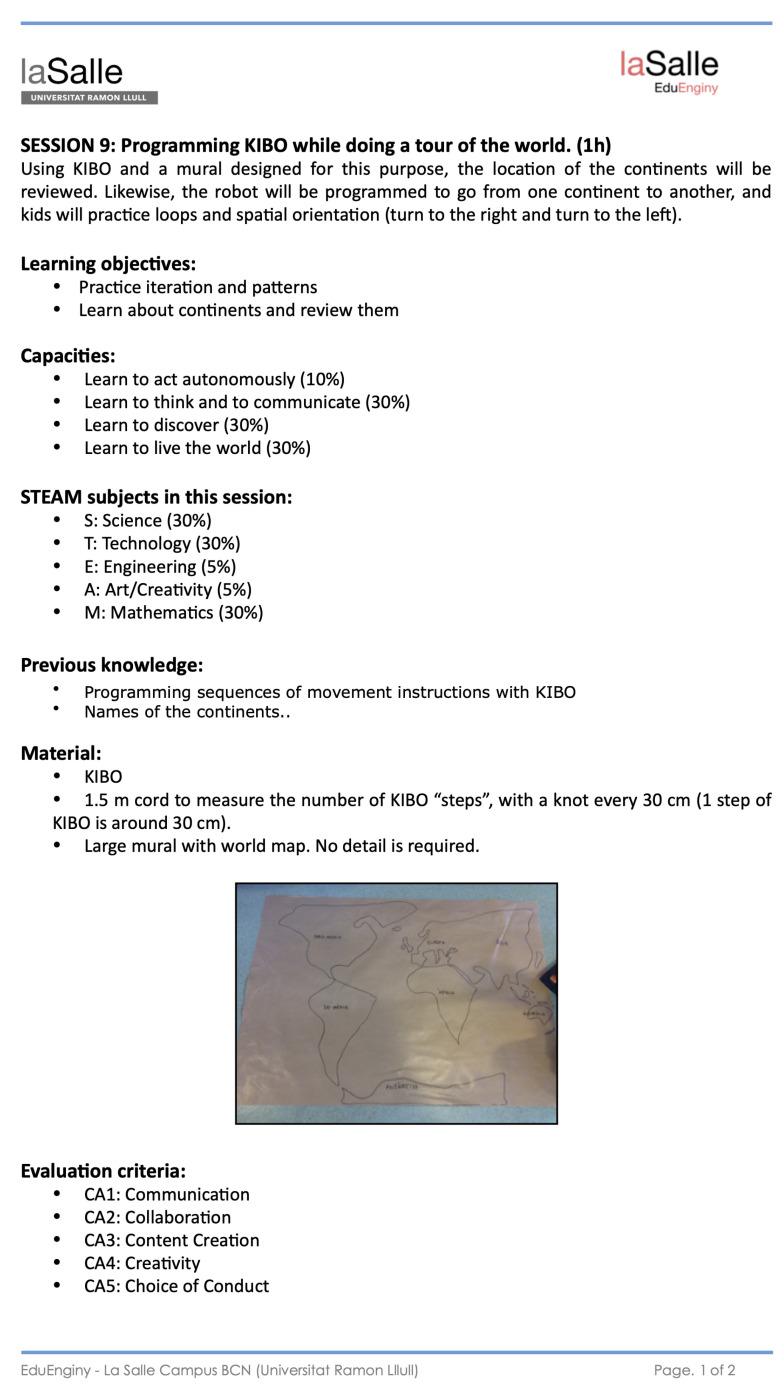

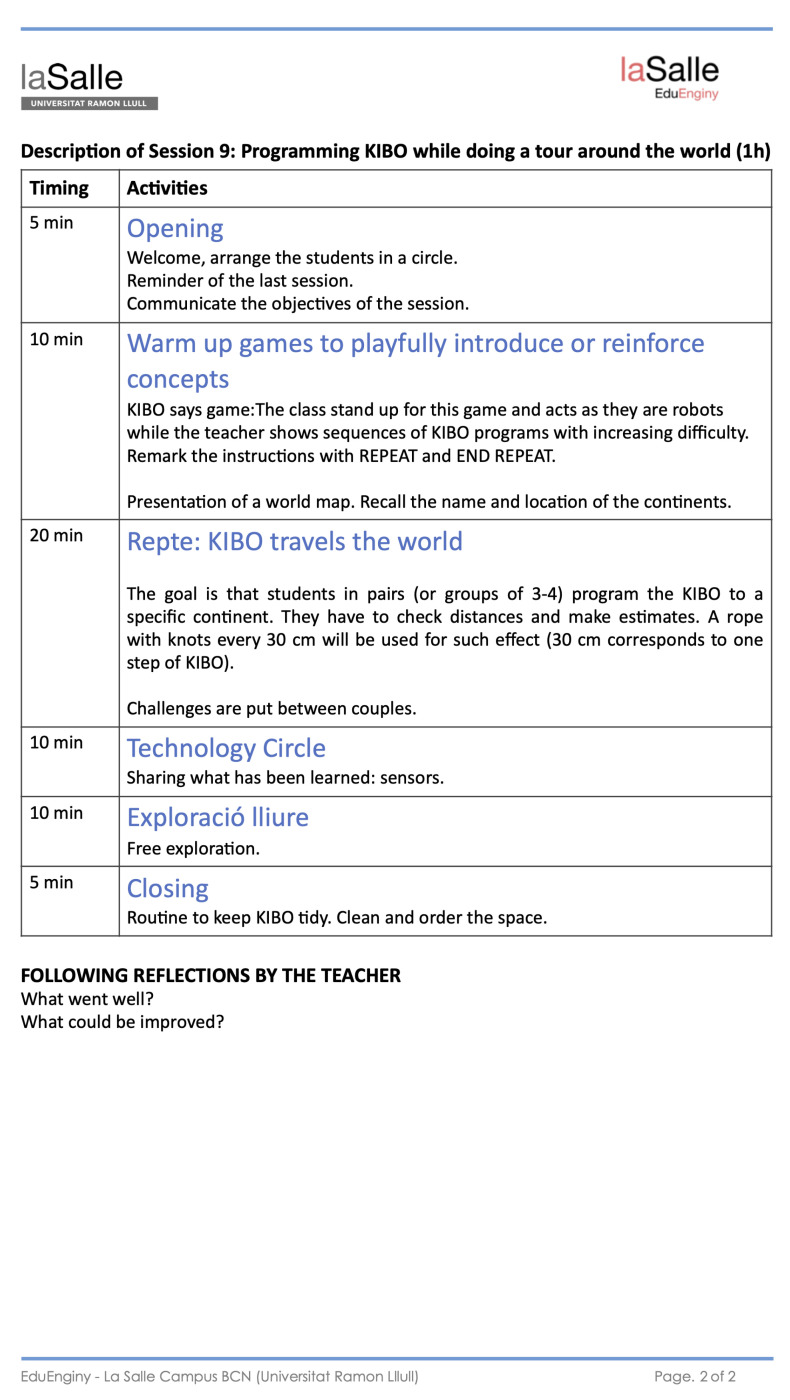
Details of a sample session of the developed curriculum.

**Figure A2:**
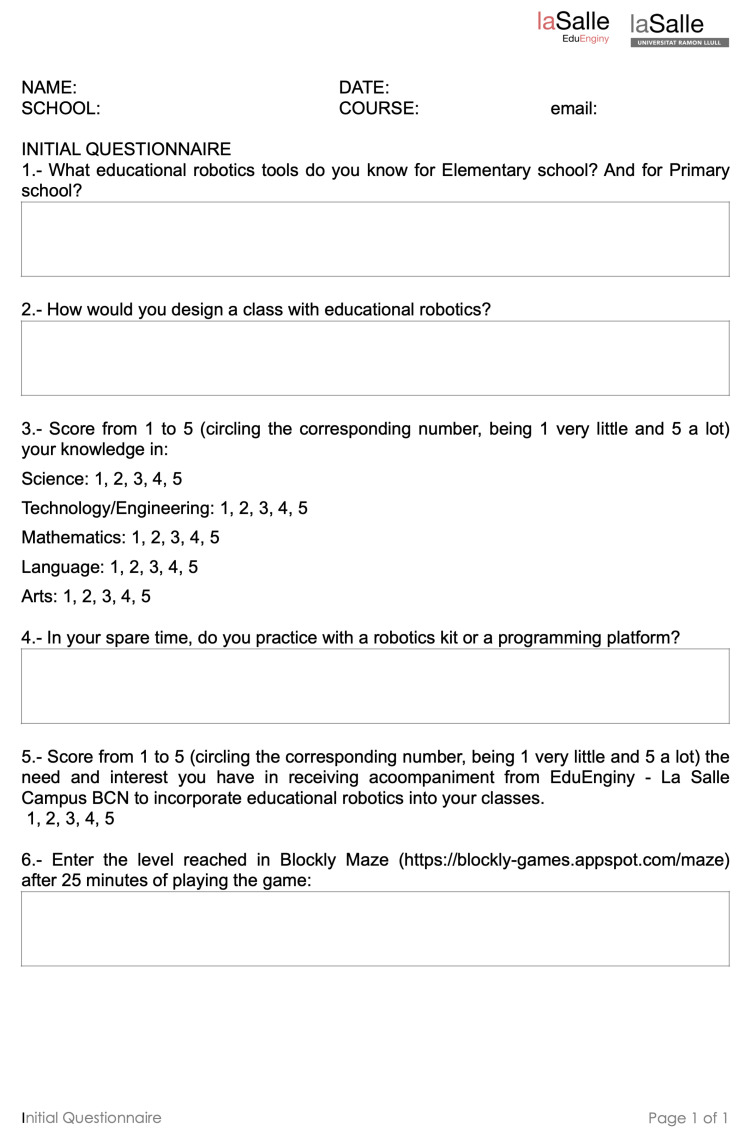
Questionnaire delivered to the teachers participating in this study before the training.

**Figure A3:**
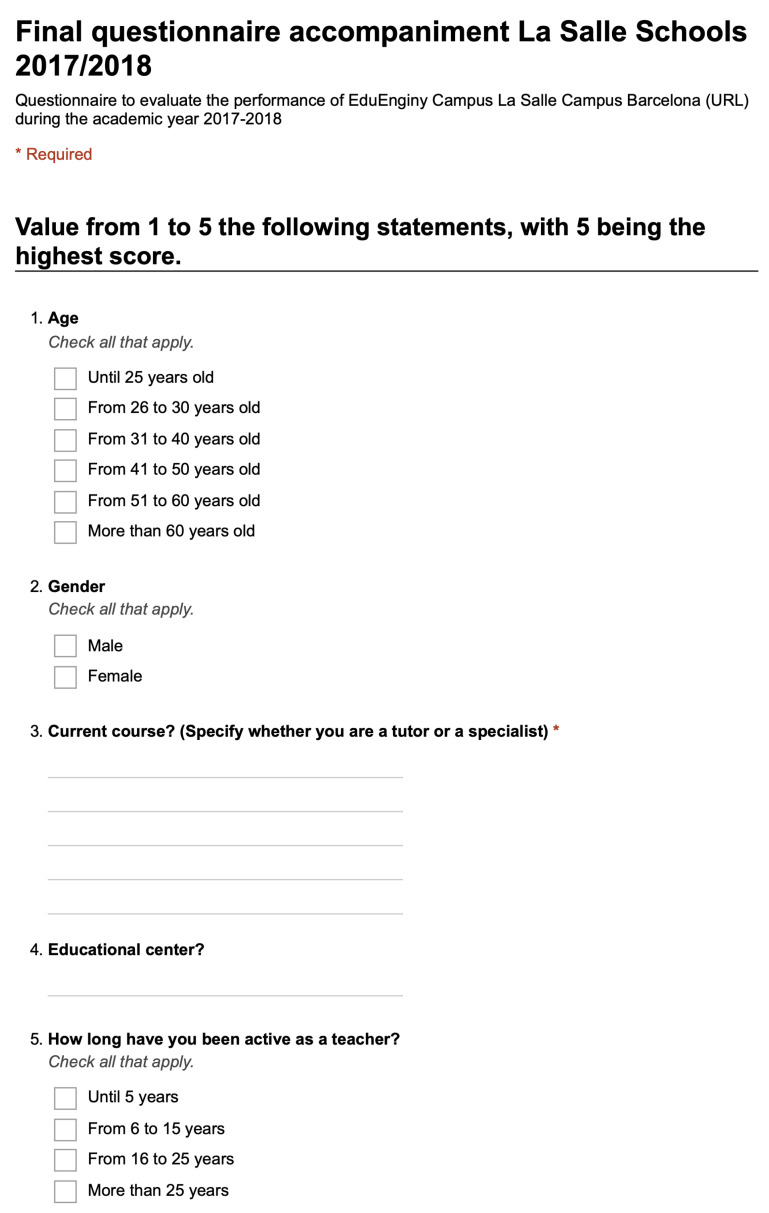

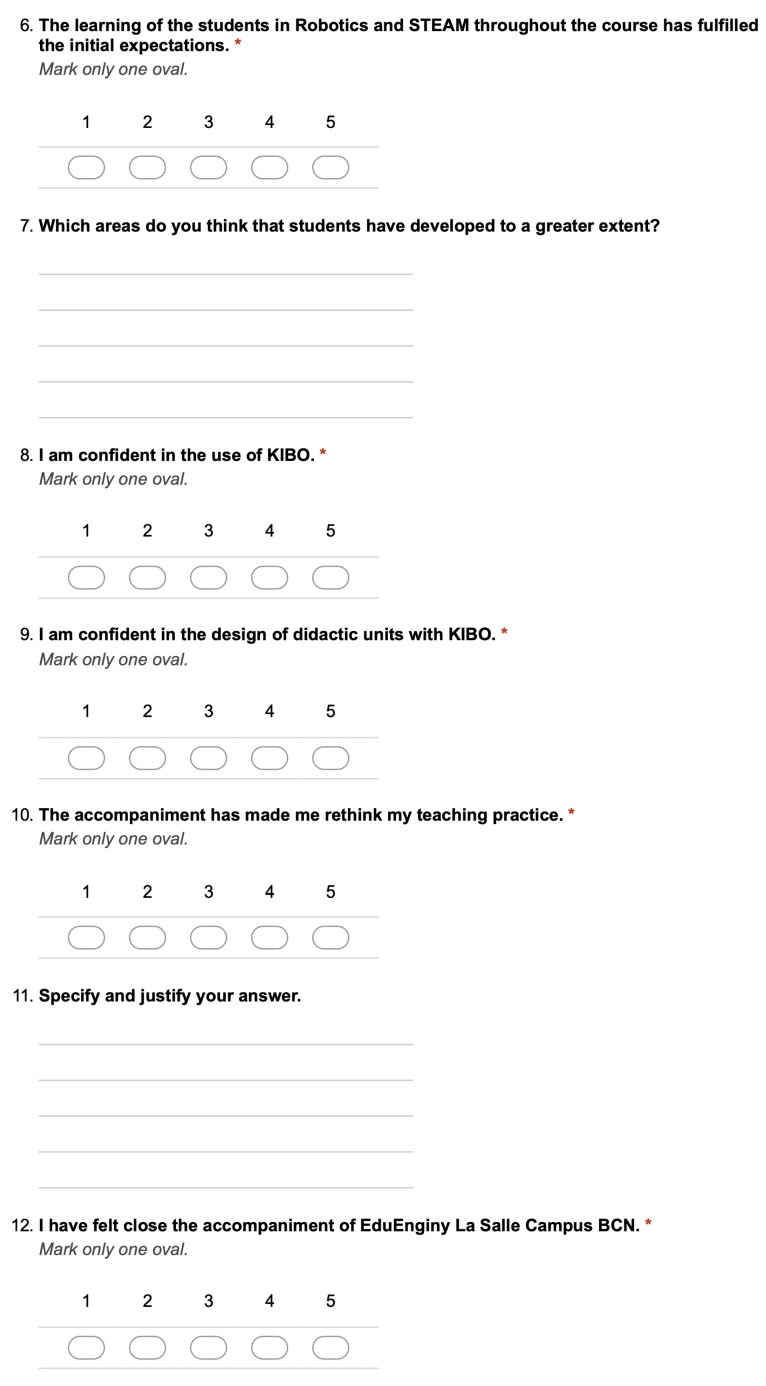

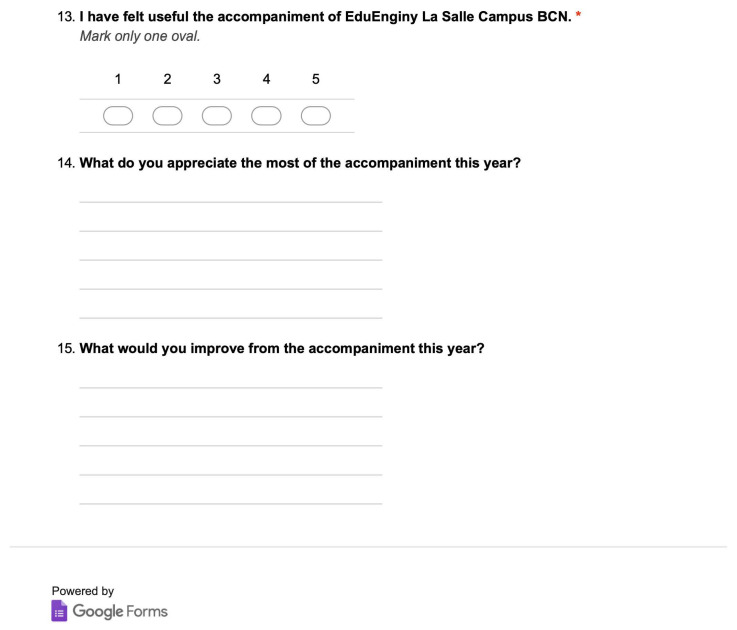
Questionnaire delivered to the teachers participating in this study after the training.

## Abbreviations

The following abbreviations are used in this manuscript:

STEAM	Interdisciplinary learning of Science, Technology, Engineering, Art, and Mathematics

## Appendix A. Details of a Sample Session of the Developed Curriculum and Web Link to the Full Curriculum

The following image (Figure A1) gives information on the learning objectives, evaluation criteria, and activities of session 9. It also contains an estimation of the contents’ percentages of Science, Technology, Engineering, Arts, Mathematics (STEAM) subjects. It has been translated into English from its original language.

The developed curriculum, with details of each of the sessions, is available in its original language (catalan) at the following link: https://drive.google.com/file/d/1V_5A954L87NJtB2daUtiIk9GoWwDaI7b/view?usp=sharing.

## Appendix B. Initial Questionnaire for the Teachers

The questionnaire presented below (Figure A2) was delivered to the teachers that took part in the study before the training. It has been translated into English from its original language.

## Appendix C. Final Questionnaire to the Teachers

The questionnaire presented below (Figure A3) was delivered to the teachers that took part in the study after the training. It has been translated into English from its original language.

## Appendix D. Evaluation Criteria Used to Evaluate the Students’ Learning

The table presented below (Table A1) lists the evaluation criteria used to evaluate the students’ learning based on the PTD (Positive Technological Development) Engagement Checklist. The PTD is an interdisciplinary approach that integrates research in applied developmental science and positive youth development with ideas from computer-mediated communication, computer-supported collaborative learning, and constructionist learning with technology [29]. It is a natural extension of the computer literacy and the technological fluency movements that have influenced the world of education but adds psychosocial and ethical components to the cognitive ones.

We used five evaluation criteria, named after five of the six sections listed in the PTD Engagement Checklist. These are communication, collaboration, content creation, creativity, and choice of conduct. We not include in this curriculum the section “community building” because the designed curriculum did not include projects to solve social or community problems.

**Table A1:** List of evaluation criteria used to evaluate the students’ learning.

Evaluation Criteria
Commmunication (CA1)
Exchanges ideas with others.
Feels comfortable seeking help and asking questions with peers and the teacher.
Collaboration (CA2)
Helps other students to understand materials.
Receives help from others and appreciates it.
Borrows or lends materials from/to one another.
Is working together with other students towards a common goal.
Content Creation (CA3)
Good process of engineering design and assembly.
Good development of computational thinking, can create a functional program for the robot/character.
Makes programs without “grammatical” errors.
Knows how to debug his/her program.
Persists in spite of obstacle or setbacks.
Creativity (CA4)
Uses a variety of materials (arts, crafts, etc.) or functions (e.g. adding a background and editing/making a character)
for his/her project.Uses technology in an unexpected way.
His/her project shows unique characteristics, i.e., it is different from everyone else’s.
Exhibits confidence and can initiate and complete a task with limited coaching.
Choice of Conduct (CA5)
Focuses on the activity and chooses to engage with it.
Follows classroom rules.
He/she is aware that his/her actions with the technology will have an impact on others.
Uses materials and resources responsibly.
Shows respectful behaviors to peers and teachers.
He/she is interested and enthusiastic about his/her project.
